# The association between antihypertensive drugs and glioma

**DOI:** 10.1038/sj.bjc.6603000

**Published:** 2006-02-21

**Authors:** M P W A Houben, J W W Coebergh, R M C Herings, M K Casparie, C C Tijssen, C M van Duijn, B H Ch Stricker

**Affiliations:** 1 Department of Neurology, St Elisabeth Hospital, PO Box 90151, 5000 LC Tilburg, The Netherlands; 2 Department of Epidemiology & Biostatistics, Erasmus MC, Rotterdam, The Netherlands; 3 Comprehensive Cancer Centre South, Eindhoven, The Netherlands; 4 Pharmo Institute, Utrecht, The Netherlands; 5 Foundation PALGA, Utrecht, The Netherlands

**Keywords:** glioma, risk, antihypertensive drugs, pharmaco-epidemiology, case–control study

## Abstract

We pursued an association between hypertension and gliomas by investigating whether antihypertensive drugs (AHD) are associated with an increased glioma risk by a population-based nested case–control study using the PHARMO database; this links dispensing records of prescription drugs to hospital discharge data on an individual basis. Pathological data were derived from the Dutch nationwide registry of histo- and cytopathology. A total of 306 glioma cases incident between 1997 and 2003 were matched to 1108 controls for year of birth, sex, geographical region and duration of follow-up. Exposure was defined as cumulative duration of AHD use and, in an alternative analysis, as cumulative dose. We estimated the magnitude of the association with conditional logistic regression analysis. Cumulative use of any AHD for more than 6 months was associated with an increased risk of glioma (OR 1.45; 95% CI 1.03–2.04). After stratification for different groups of AHD, no significantly increased risk of glioma was found for any class of AHD. After excluding a latency period of 3 years before the date of diagnosis, no association was found. In conclusion, the use of AHD seems to be associated with an increased risk of glioma, but this is probably not causal.

Although gliomas are the most common type of primary brain tumour, they are nevertheless relatively rare. In the Netherlands, world-standardised incidence rates of glioma are 6.5 per 100 000 person-years for male and 4.4 for female subjects (van der Sanden *et al*, 1998). The aetiology of glioma remains largely unclear and ionising radiation is the only established environmental risk factor, but the exposure rate is low (Ron *et al*, 1988; Karlsson *et al*, 1998). Some rare genetic disorders exist in which patients have an increased risk of glioma and a variety of other cancers (Louis and von Deimling, 1995; Melean *et al*, 2004).

Previously, we found a significantly higher prevalence of hypertension in glioma patients in a population-based case–control study in the Eindhoven Cancer Registry (Houben *et al*, 2004). We hypothesised that one possible mechanism for this association is through potentially neurocarcinogenic effects of antihypertensive drugs (AHD) or their metabolites. To test the hypothesis of a glioma-inducing effect of AHD, we conducted a population-based nested case–control study with prospectively collected automated pharmacy data, linked to morbidity and pathology data.

## MATERIALS AND METHODS

### Setting

We used the PHARMO record linkage system, a database that since 1985 has linked dispensing records of prescription drugs from a representative sample of Dutch community pharmacies to hospital discharge data on an individual basis (Herings, 1993). These pharmacies are scattered over the country and currently cover data of more than two million residents, equivalent to 12% of the Dutch population. Participants enter the database when their first prescription is filled in a PHARMO community pharmacy, and they are followed thereafter. The recorded information of each drug includes the Anatomical Therapeutic Chemical (ATC) code (WHO, 1990), the dispensing date, the quantity of the drug, the prescribed daily dose, and the estimated duration of use. Because almost all persons designate a single pharmacy to fill their drug prescriptions, dispensing histories are virtually complete (Lau *et al*, 1997).

Drug dispensing histories are linked to hospital discharge records from the Dutch Medical Register (LMR), using a validated and reliable probabilistic algorithm (Herings, 1993). The LMR comprises all hospital admissions in the Netherlands and includes dates of admission and discharge, discharge diagnoses, comorbidity and performed medical procedures. All diagnoses are coded according to the International Classification of Diseases, ninth edition clinical modification (ICD-9-CM). Since 1991, the primary diagnosis is determined by the specialist who treated the patient. Other sources of morbidity and drug exposure include hospital pharmacies, clinical laboratories and general practitioners.

### Study population and validation

As glioma is not a separate code in the ICD-9-CM, all patients with a brain tumour (ICD-9-CM code 191) in the LMR database were linked to the Dutch nationwide network and registry of histo- and cytopathology (PALGA) containing data of histological, cytological and autopsy examinations of all 16 million inhabitants in the Netherlands. We included all incident gliomas from 1 January 1997 to 31 December 2003 to include at least 12 years of potential medication histories since 1985. The index date was defined as the first admission date for a brain tumour, which was (later) confirmed to be a glioma by clinical pathology. All patients with a previous history of nonglioma cancer before the index date were excluded, identified by a history of cancer (ICD-9-CM codes 140-208), a history of radiotherapy or use of anticancer medications (ATC codes L01, L02 and L03). For each case, we took 3–4 controls from the PHARMO database, matched on geographical region, date of birth (5-year intervals), gender and duration of follow-up in the PHARMO database (3-month intervals). Duration of follow-up was defined as the difference between the date of entry in PHARMO and the index date. Controls were assigned the same index date as the cases and were part of the PHARMO population on this date. To be certain that controls were free of (yet undetected) cancer at the index date, controls with a history of cancer up to 2 years after the index date were excluded. A history of cancer in controls was identified as in the cases.

### Exposure definition

In the Netherlands, all AHD are prescription-only drugs. For all cases and controls, the complete history of filled prescriptions before the index date was obtained. The following AHD were analysed: ATC codes C02 (miscellaneous AHD), C03 (diuretics), C07 (beta-blockers), C08 (calcium antagonists) and C09 (ACE inhibitors and ATII antagonists). Exposure was calculated as the duration of use before the index date in number of days, and as the sum of dispensed defined daily doses (DDD). The DDD is the recommended standard dose for the main indication in adults, as defined by the World Health Organisation. As it is highly unlikely that occasional short-term use causes glioma, we defined a prior cutoff point of cumulative use of 6 months.

### Statistical analysis

We restricted the analyses to patients aged 30 years and over. We assessed ever use of AHD before the index date as a potential risk factor for glioma. We compared patients and controls with respect to cumulative duration of use and cumulative dose. Diuretics, beta-blockers, calcium antagonists, drugs acting on the renin–angiotensin system (ACE inhibitors, ATII antagonists) and miscellaneous AHD were analysed separately, adjusted for the other ATC classes. We also stratified for gender, age and histological subgroups of glioma.

To exclude the possibility that use of AHD was started because of symptoms in the prodromal phase of glioma, and to take into account the delay between tumour induction and diagnosis, we performed sensitivity analyses by subtracting a lag time of 3 years from the index date as performed before (Beiderbeck *et al*, 2003). *χ*^2^ statistics were used to compare proportions. Conditional logistic regression was used to estimate the association between AHD and the risk of glioma, expressed as odds ratios (ORs) and 95% confidence intervals (CI). Analyses were adjusted for residual variation in age and duration of follow-up. Since the cumulative dose can be the result of prolonged use of low-dose AHD, or of short-term high-dose AHD, we also adjusted for duration of use in the analyses for cumulative dose. In the analyses for cumulative duration of use, we additionally adjusted for mean DDD per day of use. All statistical tests were performed two-sided and statistical significance was indicated if *P*<0.05. Analyses were performed using SPSS for Windows version 12.0.1 and Statistical Analysis System version 8.2.

## RESULTS

The baseline characteristics of 306 glioma patients and 1108 matched controls aged over 30 years are shown in Table 1. The most frequent subtype was astrocytic glioma (82.3%), followed by oligodendroglioma (10.5%) and mixed glioma (4.9%), this distribution and the male excess (60.1%) being comparable with those previously reported (van der Sanden *et al*, 1998; Kleihues and Cavenee, 2000). The median follow-up time in PHARMO was 5.4 years for patients and 5.2 years for controls. The cumulative duration of AHD use was longer in patients than in controls (*P*=0.03).

In the conditional logistic regression analyses, an increased risk of glioma was found for users of any AHD (OR 1.45; 95% CI 1.03–2.04) (Table 2). When exposure was measured as cumulative dose, a nonsignificantly increased risk was found for users who received >180 DDD compared with users who received <180 DDD (OR 1.35; 95% CI 0.86–2.11). After stratification for ATC classes, none of the other risk estimates reached statistical significance, although numbers were small in some of the exposure categories (Table 2). The risk of glioma was elevated for users of miscellaneous AHD (OR 1.96; 95% CI 0.71–5.37), but not significantly.

Comparable risks were seen when exposure was measured as cumulative dose (not shown). Again, a nonsignificantly increased risk was found for users of miscellaneous AHD (OR 2.05; 95% CI 0.65–6.49). These results did not change when adjusted for the mean DDD per day of use (not shown).

In the sensitivity analyses, subtracting 3 years from the index date moved the risk estimates toward unity (Table 3), and no increased risk for users of any AHD could be shown (OR 1.08; 95% CI 0.69–1.70).

We also stratified for diagnosis (astrocytoma only, oligodendroglioma only), gender and age, and analysed for the use of any AHD. This did not change the results (not shown). In particular, no association was found for elderly men, who have the highest prevalence of hypertension (Houben *et al*, 2004).

## DISCUSSION

This is the first detailed study of the effect of AHD use on glioma risk, using prospectively collected automated pharmacy data. The higher risk of glioma for users of any AHD disappeared when a lag time of 3 years was applied; none of the different classes of AHD showed statistically significant associations.

An association between AHD-use and cancer risk is not established. There is evidence that renal carcinoma is associated with diuretic use (Grossman *et al*, 2001), although this is not generally accepted (Choi *et al*, 2005; Fryzek *et al*, 2005). Prenatal exposure to diuretics was associated with an increased risk of childhood brain tumours (Preston-Martin *et al*, 1982), which could not be confirmed by subsequent studies (Kuijten *et al*, 1990; McCredie *et al*, 1994; McKean-Cowdin *et al*, 2003). The risks of exposure to AHD in adulthood have not been thoroughly investigated although no increased risk could be shown for diuretic use (Ryan *et al*, 1992). No association was found between blood pressure and brain tumours, but numbers of cases were small, the effect of AHD was not investigated, and gliomas were not studied as a separate group (Batty *et al*, 2003).

We previously found a higher prevalence of hypertension in glioma patients (Houben *et al*, 2004). Since glioma is not known to induce clinically relevant hypertension and because hypertension is not a known risk factor for glioma, we hypothesised that the use of AHD might be the link in the observed association. The results of this study do not support such an association. Notably, potential associations disappeared in the sensitivity analyses, suggesting that hypertension is part of the prodromal signs of glioma prompting prescription of AHD though it must be conceded that such a presentation of glioma is not recognised.

Major strengths of this study are its population-base and its use of prospectively collected data on drug exposure and pathology. Pharmacy records are more complete and more reliable than medical records or patient interviews, thereby avoiding recall bias (Paganini-Hill and Ross, 1982). It has been shown that computerised pharmacy records are a reliable source of true current drug exposure (Lau *et al*, 1997), and that any misclassification is nondifferential, leading to underestimation of the true effect, rather than the reverse, in pharmaco-epidemiological studies (Lau *et al*, 1997). We considered information about glioma diagnosis to be reliable since several sources were combined, including PALGA, which is not only used for research purposes but for daily patient care. Detection bias seems unlikely as gliomas will almost always become symptomatic, regardless of medical surveillance. We therefore did not adjust for the effect of medical attention due to comorbidity, but did adjust for gender, age, duration of follow-up and geographical region. Because the PHARMO database does not contain information on lifestyle variables, we were not able to adjust for obesity, smoking or alcohol use. However, we do not expect this to be a problem since none of these factors has been associated with glioma (Wrensch *et al*, 2002).

A potential problem is that the duration of observation might have been too short to study the influence of AHD on glioma risk, possibly leading to an underestimation of risk. Furthermore, we studied five categories of AHD, based on the main mode of action. Within these categories, however, drugs can have different modes of action. For instance, ACE inhibitors and angiotensin II inhibitors are both classified in ATC group C09, but might differ in their ability to induce or promote glioma. The numbers of cases were too small to analyse these AHD separately. Similarly, for the group of miscellaneous AHD, numbers of cases were too small to draw conclusions.

In conclusion, a causal association between AHD and glioma seems unlikely, although we cannot exclude the possibility that AHD modify the occurrence of glioma. Both overall exposure and exposure to subgroups of AHD could not be clearly related to a higher risk of glioma. Some associations disappeared in the sensitivity analyses, indicating that hypertension might be a prodromal sign of glioma.

## Figures and Tables

**Table 1:** Characteristics of glioma cases and controls

	**Patients (*n*=306)**		**Controls (*n*=1108)**		
**Characteristic**	***n***	**%^a^**	***n***	**%^a^**	***P*-value**
*Age (years)*					
30–44	66	21.6	227	20.5	
45–59	115	37.6	440	39.7	
60–74	100	32.7	363	32.8	
⩾75	25	8.2	78	7.0	0.84
Median, 25th–75th percentile	56.6, 47.3–67.5		56.2, 48.0–66.4		
					
*Sex*					
Men	184	60.1	669	60.4	
Women	122	39.9	439	39.6	0.94
					
*Follow-up time in PHARMO (years)*					
0 to <1	43	14.1	164	14.8	
1 to <3	53	17.3	204	18.4	
3 to <5	50	16.3	167	15.1	
5 to <7	37	12.1	130	11.7	
7 to <9	28	9.2	104	9.4	
9 to <11	36	11.8	117	10.6	
⩾11	59	19.3	222	20.0	0.99
Median, 25th–75th percentile	5.4, 2.1–10.1		5.2, 2.0–10.1		
			
*Diagnosis*					
Astrocytoma, glioblastoma multiforme	252	82.3			
Oligodendroglioma	32	10.5			
Ependymoma	3	1.0			
Mixed glioma	15	4.9			
Glioma not otherwise specified	4	1.3			
					
*Cumulative duration of AHD use*					
No use	197	64.4	804	72.6	
<6 months	35	11.4	95	8.6	
6 months to 4 years	40	13.1	98	8.8	
⩾4 years	34	11.1	111	10.0	0.03

AHD: antihypertensive drugs.

a Percentages may deviate from 100% due to rounding.

**Table 2:** Associations between the duration of use of antihypertensive drugs and glioma, without considering a lag period of exposure

	**Patients**		**Controls**					
**Exposure**	***n***	**%**	***n***	**%**	**Adjusted OR^a^**	**95% CI**	**Adjusted OR^b^**	**95% CI**
*Any AHD*								
<6 months	232	75.8	899	81.1	Reference			
⩾6 months	74	24.2	209	18.9	1.45	1.03–2.04		
								
*Diuretics*								
<6 months	283	92.5	1034	93.3	Reference			
⩾6 months	23	7.5	74	6.7	1.13	0.68–1.89	0.87	0.50–1.50
								
*Beta-blockers*								
<6 months	268	87.6	995	89.8	Reference			
⩾6 months	38	12.4	113	10.2	1.27	0.84–1.91	1.18	0.75–1.86
								
*Calcium antagonists*								
<6 months	287	93.8	1045	94.3	Reference			
⩾6 months	19	6.2	63	5.7	1.00	0.58–1.74	0.68	0.38–1.23
								
*Ace inhibitors, ATII antagonists*								
<6 months	274	89.5	1021	92.1	Reference			
⩾6 months	32	10.5	87	7.9	1.39	0.89–2.19	1.28	0.78–2.10
								
*Miscellaneous AHD*								
<6 months	299	97.7	1097	99.0	Reference			
⩾6 months	7	2.3	11	1.0	2.40	0.91–6.37	1.96	0.71–5.37

AHD: antihypertensive drugs; OR: odds ratio; CI: confidence interval.

a Adjusted for age, gender and duration of follow-up.

b Adjusted for age, gender, duration of follow-up and for use of other types of antihypertensive drugs (ever *vs* never use).

**Table 3:** Associations between the duration of use of antihypertensive drugs and glioma, considering a lag period of exposure of 3 years

	**Patients**		**Controls**					
**Exposure^a^**	***n***	**%**	***n***	**%**	**Adjusted OR^b^**	**95% CI**	**Adjusted OR^c^**	**95% CI**
*Any AHD*								
<6 months	172	81.9	613	82.8	Reference			
⩾6 months	38	18.1	127	17.2	1.08	0.69–1.70		
								
*Diuretics*								
<6 months	196	93.3	690	93.2	Reference			
⩾6 months	14	6.7	50	6.8	1.00	0.53–1.90	0.90	0.45–1.80
								
*Beta-blockers*								
<6 months	192	91.4	669	90.4	Reference			
⩾6 months	18	8.6	71	9.6	0.87	0.49–1.53	0.81	0.44–1.50
								
*Calcium antagonists*								
<6 months	200	95.2	707	95.5	Reference			
⩾6 months	10	4.8	33	4.5	1.05	0.49–2.24	0.98	0.42–2.26
								
*Ace inhibitors, ATII antagonists*								
<6 months	197	93.8	693	93.6	Reference			
⩾6 months	13	6.2	47	6.4	0.97	0.51–1.83	1.09	0.54–2.19
								
*Miscellaneous AHD*								
<6 months	206	98.1	732	98.9	Reference			
⩾6 months	4	1.9	8	1.1	1.96	0.56–6.82	2.00	0.56–7.13

AHD: antihypertensive drugs; OR: odds ratio; CI: confidence interval.

a A lag period of exposure was considered by subtracting 3 years from the index date.

b Adjusted for age, gender and duration of follow-up.

c Adjusted for age, gender, duration of follow-up and for use of other types of antihypertensive drugs (ever *vs* never use).
